# Evaluation of anti-IL-6 monoclonal antibody therapy using murine type II collagen-induced arthritis

**DOI:** 10.1186/1476-9255-6-10

**Published:** 2009-04-15

**Authors:** Bailin Liang, Zheng Song, Bin Wu, Debra Gardner, David Shealy, Xiao-Yu Song, Paul H Wooley

**Affiliations:** 1 Department of Combination Products, Vistakon, 7500 Centurion Parkway, Jacksonville, FL 32256, USA; 2 Department of Immunobiology, Centocor, 145 King of Prussia Road, Radnor, PA 19087, USA; 3 Orthopaedic Research Institute, 929 North St. Francis, Wichita, Kansas 67214, USA; 4 Department of Biosurgicals, Ethicon, Research & Development, Route 22 West, PO Box 151, Somerville, NJ 08876, USA

## Abstract

Interleukin-6 is a multifunctional cytokine that is critical for T/B-cell differentiation and maturation, immunoglobulin secretion, acute-phase protein production, and macrophage/monocyte functions. Extensive research into the biology of IL-6 has implicated IL-6 in the pathophysiology and pathogenesis of RA. An anti-murine IL-6 mAb that neutralizes mouse IL-6 activities was tested in animal model of collagen-induced arthritis. Prophylactic treatment with anti-IL-6 mAb significantly reduced the incidence and severity of arthritis compared to control mAb treated mice. The mitogenic response of B and T cells isolated from the lymph nodes of anti-IL-6 treated mice was significantly reduced compared to cells isolated from control mAb treated mice. The overall histopathology score for paws from the anti-IL-6 treated mice was significantly reduced when compared to paws from mice treated with control mAb, including both inflammatory (synovitis and pannus) and erosive (erosions and architecture) parameters. Reduced loss of cartilage matrix components was also observed in the anti-IL-6 treated mice. Collectively, these data suggest that IL-6 plays a major role in the pathophysiology of rheumatoid arthritis, and thus support the potential benefit of anti-IL-6 mAb treatment in rheumatoid arthritis patients.

## Background

Interleukin-6 (IL-6) is a multifunctional cytokine that is critical for B-cell differentiation and maturation, immunoglobulin secretion, cytotoxic T-cell differentiation, acute-phase protein production, bone marrow progenitor stimulation, renal mesangial cell proliferation, and macrophage/monocyte functions [1]. IL-6 mediates its biological activity through binding to a receptor complex consisting of two glycoproteins, gp80 and gp130 [1]. IL-6 binding to gp80 triggers the dimerization of gp130, which results in the activation of gp130-associated Janus kinase 1 (JAK1) and subsequently signal transduction pathways.

Extensive research into the biology of IL-6 has implicated IL-6 in the pathophysiology and pathogenesis of RA [2]. High levels of IL-6 can be detected in the synovial fluid and serum in RA patients. Local expression of IL-6 may in turn stimulate leukocyte recruitment to the joint, promote osteoclast maturation and activation, potentiate aggrecanase activity to increase proteoglycan breakdown, suppress chondrocytes, and stimulate synovial proliferation, eventually culminating in joint damage [2,3]. It may also be the relevant cytokine responsible for autoimmune features in RA such as autoreactive T and B cell activation, B cell hyper reactivity and hypergammaglobulinemia [4]. Systemically, elevated IL-6 in patients with RA may induce the acute phase proteins, which contributes to the pathophysiology of some of the comorbidities of RA (ie, atherosclerosis and anemia) [5].

In preclinical models of inflammatory arthritis, deletion of IL-6 genes has resulted in protection from the induction of collagen-induced arthritis or a reduction in the disease parameters [6]. The studies reported here were designed to further elucidate the influence of IL-6 in arthritis, by examining the effects of anti-IL-6 mAb treatment in a murine model of type II collagen induced arthritis (CIA).

## Methods

### Induction and Assessment of Type II Collagen-induced Arthritis

Female DBA/1 LacJ mice 6–8 weeks of age were obtained from Jackson Labs (Bar Harbor, Maine). Mice were separated into 3 groups of 10 mice/group and injected with either an irrelevant negative mAb (Centocor CNTO1322, Radnor, PA; a non-specific rat/mouse chimeric IgG2a,k antibody that does not bind IL-6), or one of two doses of a rat anti-murine IL-6 mAb (R&D Systems, Minneapolis, MN) as described in Table 1. The two anti-IL-6 mAb treatment doses as well as the control Ab dose were selected based on data from our in vitro neutralization of IL-6-dependent 7TD-1 cell proliferation bioassay, which has been widely used [7]. Two days later after the first mAb injection, mice received an intradermal injection of 100 μg bovine type II collagen (a gift from Marie M. Griffiths, University of Utah) in Freund's complete adjuvant (FCA, Difco) at the base of the tail. Weekly intraperitoneal (IP) injections of each mAb continued for 10 weeks. Mice were weighed weekly, clinically assessed five times per week, and paw measurements were recorded three times per week. Mice were euthanized at the end of the 10-week study when lymph nodes and spleens were harvested.

**Table 1:** CIA Treatment Regimen

**Group**	**N**	**Treatment**	**Dose**
Group 1	10	control mAb	1 mg/week
Group 2	10	Anti-IL-6	1 mg/week
Group 3	10	Anti-IL-6	5 mg/week

Mice received weekly IP injections for 10 weeks. Groups 1 and 2 received 200 μL sterile PBS containing the required dose. Group 3 received 1 mL sterile PBS (500 μL on 2 consecutive days) containing the required dose of anti-IL-6.

### Clinical Assessment

Arthritic animals were clinically assessed five times per week and paw measurements were recorded three times a week for 10 weeks after disease onset. An established arthritis scoring system [8] was used to evaluate the clinical progress (Table 2). Changes in joint thickness were measured using a constant tension caliper (Dyer, Lancaster, PA), and each limb was graded giving a maximum possible clinical score of 12 per mouse. Data were collected to determine (a) if the mouse was arthritic; i.e. the presence of at least one diseased paw (b) the onset of the first visible signs of arthritis (c) the number of involved paws, which indicate changes in the progression of disease, and (d) the cumulative arthritis score in all four paws, indicating the maximum severity of arthritis. In addition to the daily clinical evaluation of arthritis, an observer (PHW) blinded to the therapeutic treatment of the animals clinically scored all mice weekly throughout the trial. Rare discrepancies between the clinical scores were resolved by the adoption of the blinded score.

**Table 2:** Arthritis Scoring System

**Arthritic score**	**Clinical Assessment**
0	Normal appearance and flexion
1	Erythema and edema
2	Visible joint distortion
3	Ankylosis detectable on flexion

### Cellular Proliferative Responses to T and B cell mitogens

In order to determine the effect of anti-IL-6 immunotherapy on lymphocyte proliferative responses, lymph node and spleen cells were stimulated with the T cell mitogen concanavalin A (ConA) and the B cell mitogen lipopolysaccharide (LPS). At the end of the study, lymph nodes and spleens were harvested from mice and single cell suspensions prepared by tissue disruption. Cells were washed, assessed for viability in trypan blue, counted and adjusted to a suspension of 2.5 × 10^6 ^cells/mL in RPMI medium supplemented with 5% fetal calf serum (FCS). Aliquots (100 μL) were dispensed into the wells of a 96-well tissue culture plate (Costar) and an equal volume of medium containing either 10 μg/mL ConA or 10 μg/mL LPS was added to appropriate wells of the tissue culture plate. Control wells (medium only) were included on the plate. Plates were incubated at 37°C in a 5% C0_2 _atmosphere for 3 days. Cell proliferation was measured by adding 20 μL of 3-(4, 5 dimethylthiazol-2-yl)-2-5 diphenyl tetrazolium bromide (MTT) solution (5 mg/mL; Sigma) to each well and incubating 6 hours at 37°C in a 5% C0_2_. The culture supernatants were replaced with 200 μL of 10% sodium dodecyl sulfate (SDS) solution, and the plates incubated at 37°C overnight. Optical density (OD) of the wells was read at 590 nm using a microplate photospectrometer (Molecular Devices). Stimulation index (SI) for each response was calculated by comparison with background proliferation (medium control). Antigen and mitogen specific responses were expressed as (OD590 [Stimulated Culture] – OD590 [Spontaneous proliferation culture]).

### Histopathological assessment

At the completion of the clinical assessment study 10 weeks post injection, limbs were removed from the mice and processed for histology. Paws were divided longitudinally at mid-line and one half flash-frozen in liquid nitrogen for RNA extraction. The remaining half was fixed in 10% neutral buffered formalin solution, decalcified for 18 days in 10% formic acid, dehydrated, and embedded in paraffin blocks. Sections were cut along a longitudinal axis, mounted and stained with hematoxylin and eosin. Specimens were cut as close to the mid line as feasible, and then sagital central samples mounted for evaluation, allowing for a consistent geographic evaluation. A minimum of 3 separate sections per specimen was evaluated in a blinded fashion. On the front limbs, all wrist and metacarpal joints were scored, while all ankle and metatarsal joints were scored on the rear paws. Digits were not evaluated, since the sectioning procedure eliminates most proximal inter-phalangeal (PIP) joints. Slides were evaluated for the presence of synovitis, pannus formation, marginal erosions, architectural changes (mostly subluxation), and overall arthritis score based on the inflammatory parameters of arthritis scoring system (Table 3).

**Table 3:** Inflammatory Parameters of Arthritis Scoring System

**Inflammatory Parameter**	**Score**	**Observation marker**
**Synovitis **(judged by the thickness of the synovial membrane)	0	less than 3 cells thick
	1	3 – 5 cells thick
	2	6 – 10 cells thick
	3	10 – 20 cells thick
	4	20 – 30 cells thick
	5	Beyond 30 cells thick

**Pannus formation**	0	No pannus formation
	1	Microvillus present
	2	Clear pannus attachment
	3	Marked pannus attachment
	4	Joint space filled by pannus
	5	Extensive pannus proliferation

**Marginal erosions**	0	No erosions visible
	1	Minor indentation in area of capsular attachment
	2	Clear erosions of cartilage
	3	Erosions extend into subchondral bone
	4	Major erosion of bone and cartilage
	5	Loss of visible cartilage and major bone loss

**Architectural changes**	0	Normal joint architecture
	1	Edematous changes
	2	Minor subluxation of articulating surfaces
	3	Major subluxation of articulating surfaces
	4	Loss of joint landmarks
	5	Complete fibrosis and collagen bridging

**Overall score**	0	Classical normal joint appearance
	1	Minor changes; consistent with remission; may be clinically normal.
	2	Moderate inflammatory disease
	3	Major inflammatory disease
	4	Destructive, erosive arthritis
	5	Destructive, erosive arthritis with major bone remodeling.

### Cartilage and Bone Matrix degradation

Serial sections of joints were stained for cartilage matrix components using 2 histochemical stains, Toluidine Blue and Aldehyde Fuchsia. Slides were evaluated for loss of matrix components using the scoring system in Table 4.

**Table 4:** Matrix Degradation Scoring System

**Toluidine Blue**	
0	No visible Toluidine Blue staining
1	Very weak Toluidine Blue staining only in the deep cartilage
2	Weak Toluidine Blue staining
3	Moderate Toluidine Blue staining
4	Some loss of staining from the superficial cartilage
5	Normal Toluidine Blue staining

**Aldehyde Fuchsia**	

0	No visible Aldehyde Fuchsia staining
1	Very weak Aldehyde Fuchsia staining only in the deep cartilage
2	Weak Aldehyde Fuchsia staining
3	Moderate Aldehyde Fuchsia staining
4	Some loss of staining from the superficial cartilage
5	Normal Aldehyde Fuchsia staining

### Serum amyloid A (SAA) analysis by enzyme-linked immunosorbent assay (ELISA)

SAA levels were determined by ELISA (Biosource, Camarillo, CA) according to the manufacturer's recommendations. Briefly, serum samples were diluted 1:200 in assay diluent and incubated with conjugated anti-mouse SAA antibody. Following multiple plate wash cycles, the substrate tetramethylbenzidine was added, and the optical density of the samples was read at OD_450 nm_. The results were analyzed to determine SAA using a four-parameter fit of the standard curve provided to determine sample levels of SAA.

### Real-time PCR analysis of Paw RNA

Paws were harvested as described above and tissues homogenized with a Polytron RT 2000 in 7.5 M guanidium-HCl for 3 minutes, followed by the addition of sodium lauryl sarcosinate to a final concentration of 0.5%. After centrifugation to remove debris, and the addition of 2 M potassium acetate and 1 M acetic acid, RNA was precipitated by the addition of cold absolute ethanol. Total RNA was isolated and processed using the RNAzol method (Tel-Test, Friendswood, TX) according to the manufacturers instructions. The quantity and purity of RNA was determined by absorbance on a spectrophotometer (Beckman Instruments, Fullerton, CA.) at 260 nm and 280 nm. Samples with ratios > 1.7 were accepted for analysis. To analyze the gene expression real time reverse-transcription polymerase chain reactions (RT-PCR) were performed and the gene activity of OPG and TNFα examined. cDNA was reverse transcribed from 0.5 μg of total RNA in a 20 μl reaction mixture containing 1× PCR buffer, 500 μM each of deoxynucleotide triphosphates (dNTP), 0.5 U/μl of RNAse inhibitor, 2.5 μM random hexamers, 5.5 mM MgCl_2_, and 1.25 U/μl of reverse transcriptase (Perkin Elmer, CT). The reaction mixture was incubated in a Thermal Cycler (Perkin Elmer, CT) at 25°C for 10 minutes, 48°C for 25 minutes followed by 95°C for 5 minutes. Real time PCR was performed according to manufacturer's instructions. To standardize target gene level with respect to variability in quality of RNA and cDNA, we used glyceraldehyde-3-phosphate dehydrogenase (GAPDH) transcripts, a housekeeping gene, as an internal control. Reaction mixtures of 25 μl included 12.5-μl of 2× SYBR^® ^Green Master Mix and target gene primer pairs (at 400 nM final concentration) and 2 μl cDNA. The sequences of the primers were purchased from Clontech, CA. All reagents were from Perkin Elmer/Applied Biosystems. The reactions were run in MicroAmp optical 96-well reaction plates with MicroAmp optical caps for 40 cycles (95°C/15-seconds, 60°C/1-min) in the ABI Prism 7700 Sequence Detector (PE-Applied Biosystems, Foster City, CA) and the fluorescent signals were recorded dynamically. Normalization and analysis of the reporter signals (ΔRn) at the threshold cycle was recorded by the machine built-in software, and target gene copies were calculated against the regression of the standard curve.

### Statistical analysis

Appropriate statistical comparisons (Kruskal-Wallis ANOVA and Mann-Whitney U tests) were performed to assess the influence of anti-IL-6 on disease progression (based on cumulative arthritis score) and histological assessment. Appropriate statistical comparisons were also performed to assess the influence of treatment on the cellular immune response with respect to the T cell mitogen ConA and the B cell mitogen LPS. One-way ANOVA tests were performed to assess the influence of IL-6 antibody on histopathological features of disease and loss of cartilage matrix proteins. Post-hoc LSD scores were used to assess differences between the individual groups, and compared both treatment groups to control, and the two treatment groups together. P values < 0.05 were considered statistically significant.

## Results

### Influence of IL-6 antibody on collagen-induced arthritis

**Figure 1:**
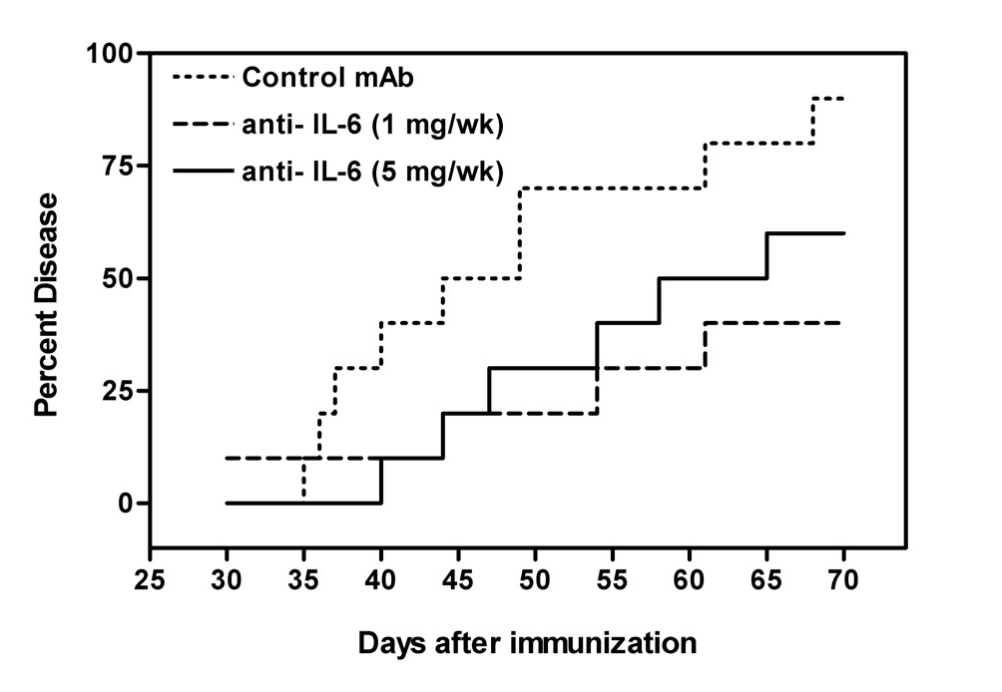
**Anti-IL-6 decreased incidence of CIA**. DBA/1 LacJ mice were dosed IP with anti-IL-6 antibody (1 mg or 5 mg/mouse) or irrelevant control antibody (1 mg/mouse) two days prior to injection with collagen and weekly IP for 10 weeks (n = 10 mice/group). Percent disease represents the percentage of mice/group with clinical signs of arthritis (having at least one diseased paw). Statistical differences between the groups were determined using the Kruskal-Wallis test for multiple samples (SPSS Inc, Chicago, IL).

Intraperitoneal administration of IL-6 antibody was observed to exert a suppressive effect on the development of CIA (Figure 1). The 1 mg/week dose was more successful in the prevention of the final incidence of disease development (week 10, p < 0.02) while the reduced incidence observed using 5 mg/week did not achieve statistical significance (p = 0.13). However, from Day 49 to Day 53, both treatment regimes did result in a statistically significant decrease in the incidence of collagen arthritis (p < 0.05).

**Figure 2:**
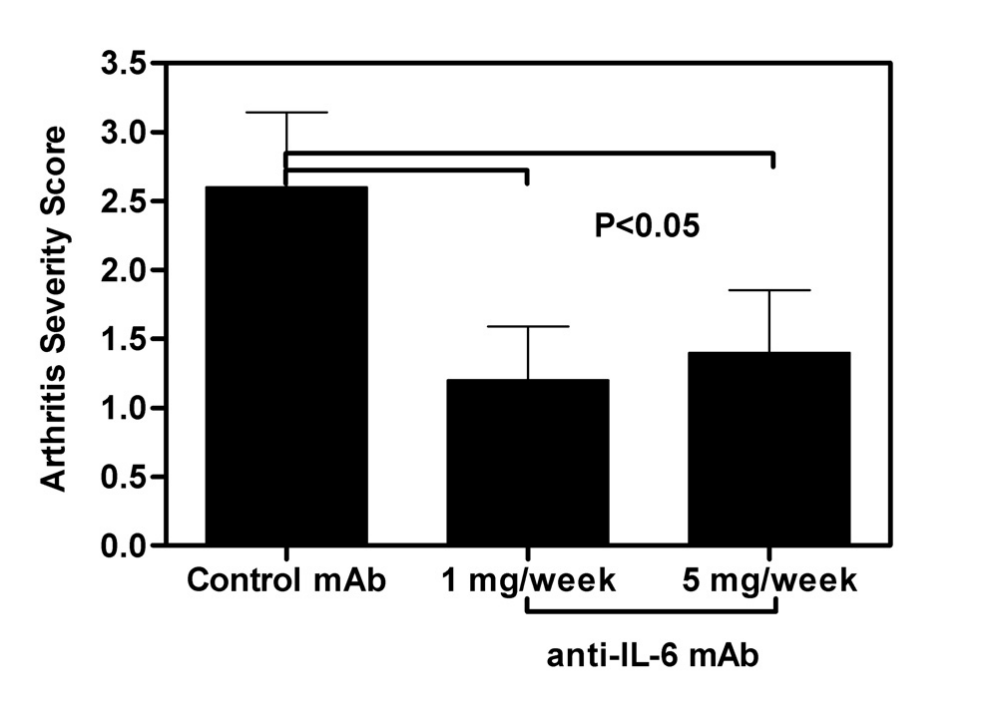
**Anti-IL-6 reduced severity of CIA**. DBA/1 LacJ mice were dosed with anti-IL-6 antibody (1 mg or 5 mg/mouse) or irrelevant control antibody (1 mg/mouse) two days prior to injection with collagen and weekly for 10 weeks. Bars represent observed arthritis severity score (mean ± SEM) at the end of the study (day 70), based on the arthritis scoring system (Table 2). There was a significant difference (p < 0.05) between the control mAb and both anti-IL-6 mAb doses. Statistical differences between the groups were determined using ANOVA (SPSS Inc, Chicago, IL).

Anti-IL-6 was efficacious at both the 1 mg/week and 5 mg/week doses in reducing the severity of collagen-induced arthritis (Figure 2). The mean arthritic score in control mice (2.9) was significantly higher than the mean score of 1.2 observed in mice treated with 1 mg/week anti-IL-6 (p = 0.016) or the mean score of 1.4 seen in mice treated with 5 mg/week anti-IL-6 (p = 0.03).

**Figure 3:**
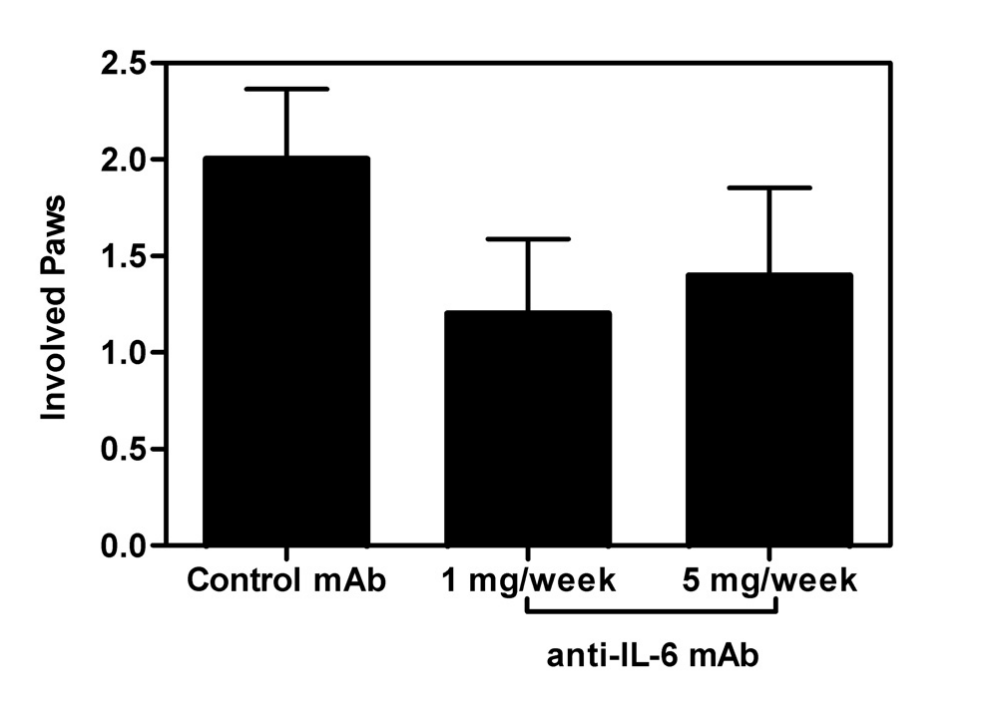
**Anti-IL-6 inhibited disease progression**. DBA/1 LacJ mice were dosed with anti-IL-6 antibody (1 mg or 5 mg/mouse) or irrelevant control antibody (1 mg/mouse) two days prior to injection with collagen and weekly for 10 weeks. Bars represent number of involved paws (mean ± SEM) in each mouse at the end of the study (day 70). Statistical differences between the groups were determined using ANOVA (SPSS Inc, Chicago, IL).

In this animal model, the disease is usually initiated in one paw and then spreads to other paws, which is an indication of disease progression. In the control group, the mean number of involved paws at the conclusion of the trial was two. Anti-IL-6 therapy exhibits a trend towards the suppression of disease progression, as the majority of treated animals did not progress to arthritis in two paws (Figure 3). However, the absolute number of paws affected in the IL-6 treated groups was not statistically different from the control group.

### Immunological assessment of proliferative responses of spleen and lymph node cells

**Figure 4:**
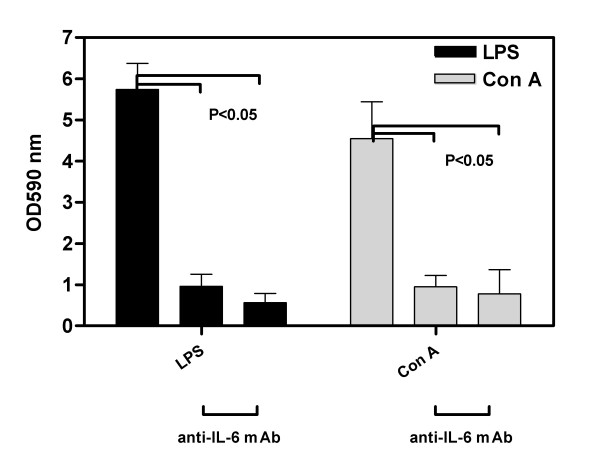
**Immunotherapy with anti-IL-6 suppressed *in vitro *lymph node cell responses to mitogens**. DBA/1 LacJ mice were dosed IP with anti-IL-6 antibody (1 mg or 5 mg/week) or irrelevant control antibody (1 mg/week) two days prior to injection with collagen and weekly IP for 10 weeks. Lymph nodes were harvested at the end of the study and isolated lymphocytes cultured with ConA or LPS for 3 days. Cell proliferation was measured using MTT. Bars represent mean ± SEM OD_590 _as measured in the assay. There was a significant difference (p < 0.05) between the control mAb and both anti-IL-6 mAb doses for both ConA and LPS mitogens. Statistical differences between the groups were determined using ANOVA (SPSS Inc, Chicago, IL).

Immunotherapy with anti-IL-6 resulted in a significant depression of *in vitro *lymph node cell responses to mitogens (Figure 4). Proliferation following stimulation with either LPS or Con A was reduced to a highly significant extent (p < 0.001) in mice treated with either 1 mg/week or 5 mg/week of anti-IL-6 antibody compared to control. The T cell mitogen response was diminished in Group 3 (5 mg IL-6 antibody) in the spleen with respect to both the control and Group 2 (1 mg IL-6 antibody), while the LPS response was unaffected (data not shown).

### Influence of IL-6 antibody on the inflammatory histopathology of CIA

**Figure 5:**
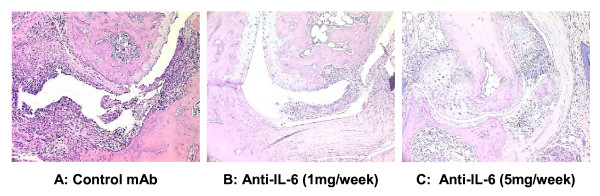
**Anti-IL-6 antibody reduced the inflammatory histopathology of CIA**. DBA/1 LacJ mice were dosed with irrelevant control antibody (A) or anti-IL-6 antibody (1 mg/week (B) or 5 mg/week (C)) for 10 weeks. Mice were sacrificed and joint specimens prepared for histopathological assessment as described in materials and methods. Photomicrographs are 200×.

Intraperitoneal administration of 1 mg/week or 5 mg/week of IL-6 antibody during the course of CIA had a beneficial effect on the terminal histopathology of CIA. Typical arthritis seen in mice from the control mAb group is shown in Figure 5A. The disease was moderately severe, with erosions extending through the hyaline cartilage and deep into the subchondral bone. The parameters of inflammation were marked, with notable synovitis and pannus formation. In contrast, disease seen in animals treated with 1 mg of IL-6 antibody was significantly ameliorated (Figure 5B). The synovitis was less extensive, and erosions were confined to the marginal area. In general, the articulating surfaces of the cartilage were spared, and joint spaces appeared essentially normal. This pattern was consistent in mice treated with 5 mg/week of IL-6 antibody (Figure 5C). In these animals, when arthritis was present, the synovitis was slightly more severe than that observed in the 1 mg/week group, but this difference was not marked. Overall, arthritis was less severe in animals treated with IL-6 antibody.

**Figure 6:**
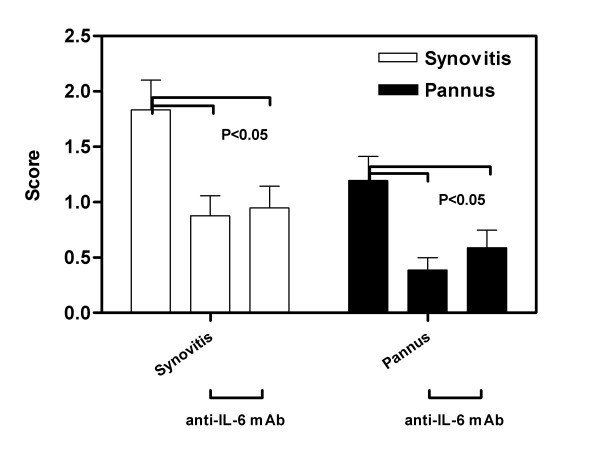
**Anti-Il-6 treated mice had reduced synovitis and pannus scores**. DBA/1 LacJ mice were dosed with anti-IL-6 antibody (1 mg or 5 mg/week) or irrelevant control antibody in CIA model for 10 weeks. Mice were sacrificed and joint specimens prepared for histopathological assessment as described in materials and methods. Specimen slides were evaluated using the scoring system from Table 3. Bars represent the mean (± SEM) of the inflammatory score for each parameter for all 4 paws of each mouse in the group. There was a significant difference (p < 0.005) between the control mAb and both anti-IL-6 mAb doses for both synovitis and pannus. Statistical differences between the groups were determined using ANOVA (SPSS Inc, Chicago, IL).

Quantification of the histology scores for all the mice under study revealed that both synovitis and pannus formation were significantly reduced (p < 0.005) in mice receiving anti-IL-6 compared with control mAb animals (Figure 6). There were no significant differences between mice receiving 1 mg/week and 5 mg/week IL-6 mAb.

### Influence of IL-6 antibody on the erosive histopathology of collagen-induced arthritis

**Figure 7:**
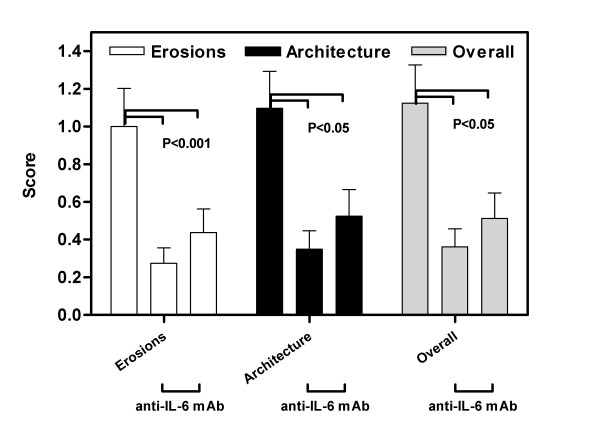
**Anti-IL-6 therapy reduced the erosive parameters of CIA**. DBA/1 LacJ mice were dosed with anti-IL-6 antibody (1 mg or 5 mg/mouse) or irrelevant control antibody in the CIA model for 10 weeks. Mice were sacrificed and joint specimens prepared for histopathological assessment as described in materials and methods. Specimen slides were evaluated using the scoring system from Table 3. Bars represent the mean (± SEM) of the inflammatory score for each parameter for all 4 paws of each mouse in the group. There was a significant difference (p < 0.001) between the control mAb and both anti-IL-6 mAb doses for all parameters. Statistical differences between the groups were determined using ANOVA (SPSS Inc, Chicago, IL).

The evaluation of the effect of anti-IL-6 on the erosive parameters of collagen arthritis (erosions and joint architecture) revealed that the therapy was successful in reducing these features (Figure 7). The reduction of erosive changes was highly significant (p < 0.001) when the anti-IL-6 treated mice were compared with control mAb animals. However, there was no significant difference between mice treated with different doses of anti-IL-6. The overall histological score also showed a highly significant reduction in both groups of mice treated with anti-IL-6.

### Influence of IL-6 antibody on loss of cartilage matrix components

**Figure 8:**
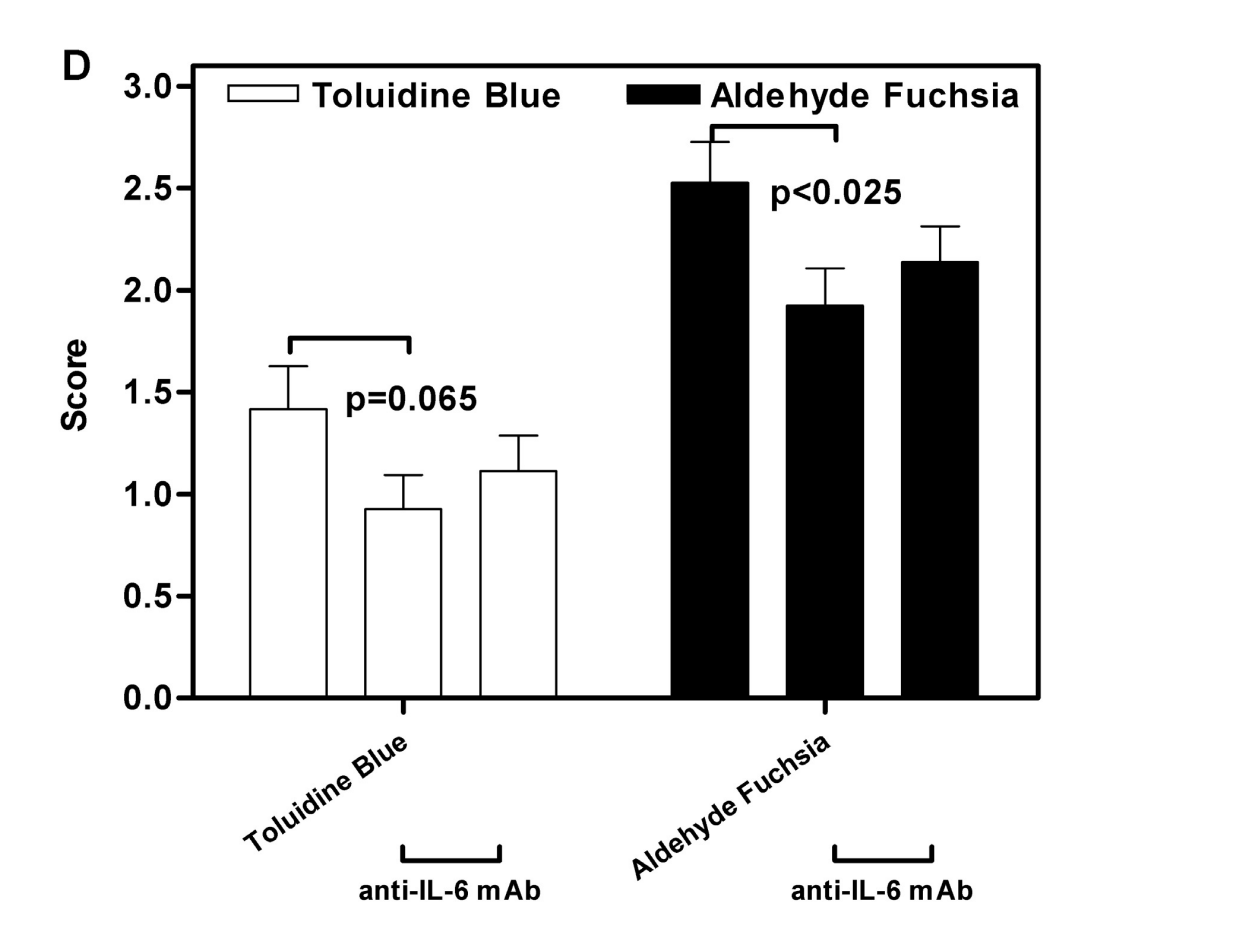
**Anti-IL-6 immunotherapy reduced the loss of cartilage matrix components in CIA**. Representative images of cartilage matrix component loss during collagen-induced arthritis in mice treated with control antibody (A), 1 mg/week anti-IL-6 (B) or 5 mg/week anti-IL-6 (C). Photomicrographs are 200×. Image analysis of the staining densities was conducted to quantify the matrix protein loss in photomicrographs (D). Bars represent the mean (± SEM) value of staining density based on the matrix degradation scoring system defined in Table 4. Statistical differences between the groups were determined using ANOVA (SPSS Inc, Chicago, IL).

Immunotherapy with anti-IL-6 was seen to exert a protective effect upon the loss of cartilage matrix components during collagen arthritis (Figure 8). Representative photomicrographs of sections stained with Toluidine Blue are shown. In mice from the control group (Figure 8A), a marked loss of matrix components may be seen, particularly at the cartilage articulating surfaces and the regions of inflammatory erosion. The staining reveals a high level of chondrocyte necrosis, with empty lacunae observed in areas of high inflammatory activity. In contrast, mice receiving anti-IL-6 antibody therapy exhibited less matrix component loss. Immunotherapy at 1 mg/week (Figure 8B) retarded matrix protein loss at the articulating surface, although areas of chondrocyte death were still visible. Staining was denser, and suggested that overall cartilage health was improved. In mice receiving anti-IL-6 at 5 mg/week, a similar pattern of reduced matrix protein loss was observed, and the articulating surface in several mice appeared well preserved (Figure 8C). Image analysis of the staining densities was conducted to quantify the matrix protein loss (Figure 8D). Mice receiving 1 mg/week exhibited a significant reduction in staining loss using aldehyde fuchsia (p < 0.025) and approached significance for a reduction in staining loss using Toluidine Blue (p = 0.065). Reductions in cartilage matrix loss observed in mice receiving 5 mg/week were not significant compared to control mice.

### Influence of IL-6 antibody on serum SAA levels

**Figure 9:**
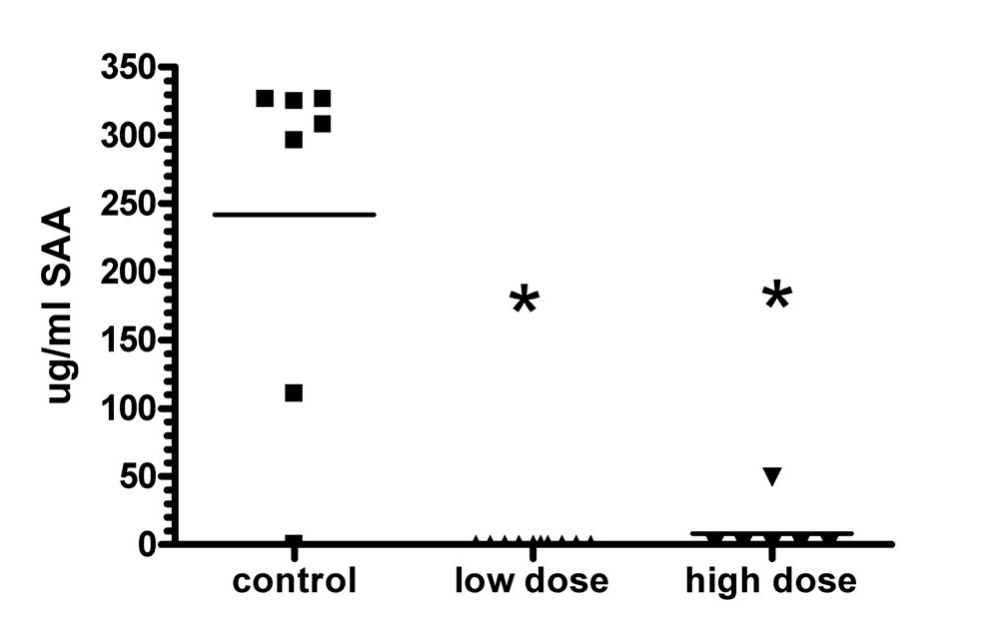
**Anti-IL-6 mAb inhibited SAA production in CIA**. DBA/1 LacJ mice were dosed with anti-IL-6 antibody (1 mg or 5 mg/mouse) or irrelevant control antibody in the CIA model for 10 weeks. Serum samples were collected at final harvest and SAA levels were measured by ELISA. * p < 0.05 versus isotype control mAb-treated groups. Statistical differences between the groups were determined using ANOVA (SPSS Inc, Chicago, IL).

SAA is an acute phase protein whose production is induced by IL-6. Intraperitoneal administration of 1 mg/week or 5 mg/week of IL-6 antibody during the course of CIA significantly reduced SAA in the serum (Figure 9).

### Influence of anti-IL-6 treatment on TNFα and osteoprotegerin expression in paws

**Figure 10:**
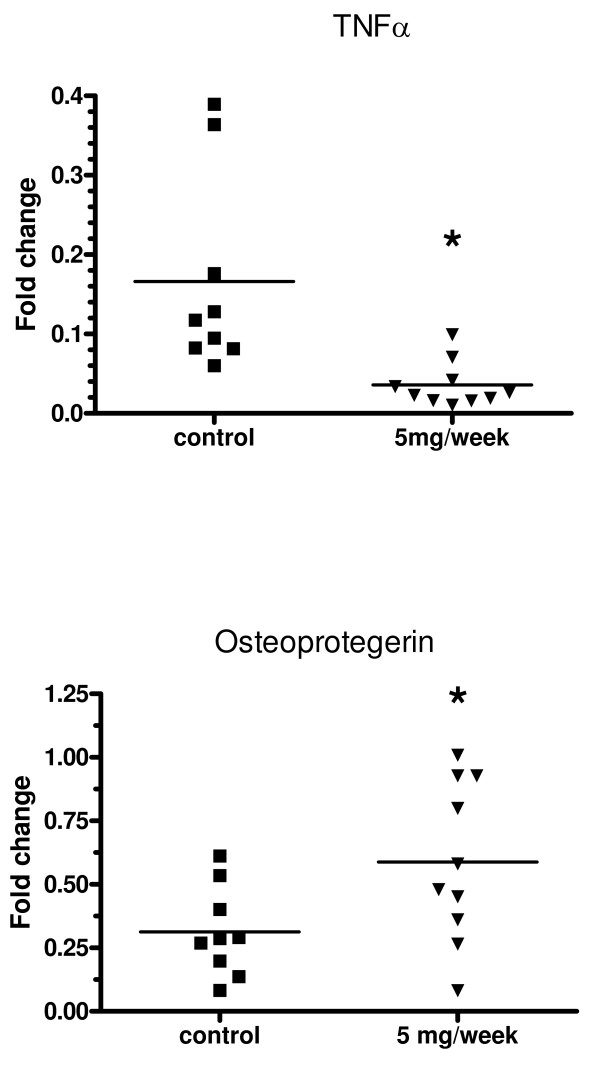
**Anti-IL-6 mAb inhibited TNFα and OPG expression in CIA**. DBA/1 LacJ mice were dosed with anti-IL-6 antibody (5 mg/mouse) or irrelevant control antibody in the CIA model for 10 weeks. Paws were collected at final harvest and RNA was purified. cDNA was prepared and analyzed using real-time PCR. * p < 0.05 versus isotype control mAb-treated groups. Statistical differences between the groups were determined using ANOVA (SPSS Inc, Chicago, IL).

Intraperitoneal administration of 5 mg/week of anti-mIL-6 resulted in a significant decrease in TNFα expression, and a significant increase in osteoprotegerin expression in mouse paws determined by real-time PCR (Figure 10).

## Discussion

An anti-murine IL-6 mAb that neutralizes mouse IL-6 activities was tested in an animal model of CIA. Prophylactic treatment with anti-IL-6 mAb significantly reduced the incidence and severity of arthritis as compared to control mAb treatment. The overall histopathology score for paws harvested from anti-IL-6 treated mice was significantly reduced when compared to paws harvested from mice treated with control mAb, including both inflammatory (synovitis and pannus) and erosive (erosions and architecture) parameters. A linear dose response relationship was not observed in the study, with a similar efficacy observed between the 5 mg/week and the 1 mg/week doses during the majority of the trial. This suggests that a maximum threshold of anti-arthritic activity may be achieved by anti-IL-6 at or even below the 1 mg/week dose level. Thus the current study cannot provide pharmacokinetics on the efficacy of anti-IL-6 therapy, beyond the observation of a marked reduction in disease parameters. The mitogenic response of B and T cells isolated from the lymph nodes of anti-IL-6 treated mice was significantly reduced compared to cells isolated from control mAb treated mice. These data suggest that IL-6 plays a major role in the pathophysiology of rheumatoid arthritis, and thus indicate that anti-IL-6 mAb may be beneficial in the therapy for RA patients.

IL-6 mediates both acute and chronic phases of inflammatory responses, therefore involved in early and late stages of inflammation. IL-6 plays a critical role in stimulating T/B cell proliferation and differentiation. Circulating serum IL-6 has been shown to be elevated in multiple inflammatory diseases including RA, systemic juvenile idiopathic arthritis, systemic lupus erythematosus, ankylosing spondylitis, psoriasis and Crohn's disease [9]. A clear correlation between IL-6 levels and markers of inflammation was established in patients with these diseases. IL-6-mediated chronic inflammatory proliferation results in the typical plasmacytosis and hyperplasia of synovial cells in the joints of rheumatoid arthritis patients as well as in control animals in our study. IL-6 dose not only initiate and maintain inflammation, but also perpetuate inflammatory responses through manipulation of the various cell types involved in inflammation. Our current study demonstrated that anti-IL-6 mAb treatment effectively suppressed IL-6 bioactivities and subsequent disease development in a murine RA model.

IL-6 has been reported to stimulate endothelial cell production, resulting in the release of IL-8 and monocyte chemoattractant protein, expression of adhesion molecules, and recruitment of leukocytes to inflammatory sites [2]. Furthermore, IL-6 can stimulate synoviocyte proliferation and osteoclast activation, resulting in synovial pannus formation [2]. With the help of IL-1, IL-6 can increase production of matrix metalloproteinases, which ultimately contributes to joint and cartilage destruction [10]. Synovitis and pannus formation were significantly reduced in the anti-IL-6 mAb treated mice as compared to that of the control mAb treated mice in the current study, indicating the efficacy of the anti-IL-6 mAb in suppressing histological degradation in this RA model. IL-6 receptor subunit gp130 transgenic mice that have excess spontaneous IL-6 signaling have been reported to develop a joint disease that is similar to RA which is triggered by lymphocyte activation and accompanied by autoantibody formation [11]. However, deletion of IL-6 gene has resulted in protection from the induction of collagen-induced arthritis or a reduction in the disease parameters [6]. Intra-articular administration of IL-6 into the joints of IL-6-deficient mice did not lead to arthritis, but a soluble IL-6/IL-6R fusion protein caused joint swelling and other symptoms of arthritis [12]. Other animal studies have demonstrated that IL-6 is critical in the development of experimental arthritis. The development of CIA in anti-mouse IL-6R antibody (MR16-1)-treated mice was significantly inhibited [13]. Similar results have also been reported in antigen-induced arthritis in mice [14] and in SKG mice that spontaneously develop autoimmune arthritis [15]. In summary, these results suggest that reduced levels of IL-6 protect against development of inflammatory arthritis, while excess IL-6 signaling contributes to disease development. Our currently study confirms this finding.

Validation of the IL-6 signaling pathway as a target in RA has also been established by the clinical efficacy in multicenter trials of tocilizumab, a humanized anti-IL-6 receptor antibody [16], and in a pilot study of B-E8, a murine anti-IL-6 monoclonal antibody (mAb) [17]. Tocilizumab treatment resulted in rapid improvements in arthritis and fever in an open-label study in children with systemic-onset juvenile idiopathic arthritis. Tocilizumab also has demonstrated inhibition of radiographic progression in one trial, further supporting the tenet that blocking the IL-6 pathway interrupts inflammatory disease processes in RA. We believe both of the anti-IL-6 and anti-IL-6R mAbs will block IL-6 signaling and the inflammation mediated by IL-6, however, these two approaches might be different in terms of relevant indications, efficacy, and toxicity profile, especially in chronic diseases.

IL-6 is required for CIA development, and the control of osteoclast formation, activation and apoptosis is mediated in part by OPG, a secreted TNF receptor family member that functions as a decoy receptor for RANKL, inhibiting osteoclast formation and activation, and accelerating apoptosis. Studies have shown that OPG reduces osteoclast numbers and prevents bone erosion in CIA. It has also been shown that decreased levels of IL-6 and increased levels on OPG lead to a protective effect on bone destruction in CIA. In our study, anti-IL-6 mAb treatment decreased TNFa levels and increased OPG levels in paws of mice after induction of CIA. The mechanism of action of IL-6 may have been through a decrease in the activity of TNFa, a potent osteoclastogenic cytokine that is known to be central to the pathogenesis of RA, and an increase in OPG, leading to decreased osteoclast formation and activity.

## Conclusion

Taken together, our study confirms the strong link between IL-6 and autoimmune arthritis in an animal model. Biologic blocking IL-6 ligand has led to the amelioration of RA symptoms and disease activities. We believe targeting the ligand might be different from targeting the receptor in terms of efficacy and toxicity as we gradually learn from the other biologic therapeutics such as TNFα and TNFα receptor mAbs. Our study represents an alternative approach to block the IL-6 signaling.

## Competing interests

The authors declare that they have no competing interests.

## Authors' contributions

ZS, BW, and PHW carried out the studies and participated in the design of the study and performed the statistical analysis. BL and PHW drafted the manuscript. DG carried out the SAA ELISA and real time RT-PCR assays. XS and PW conceived of the study, and participated in its design and coordination. DS reviewed and edited the manuscript. All authors read and approved the final manuscript.
